# Seasonality in physical activity and walking of healthy older adults

**DOI:** 10.1186/s40101-015-0071-5

**Published:** 2015-10-02

**Authors:** Tasuku Kimura, Hiromitsu Kobayashi, Eijun Nakayama, Wataru Kakihana

**Affiliations:** University Museum, The University of Tokyo, 7-3-1, Hongo, Bunkyo, Tokyo, 113-0033 Japan; Ishikawa Prefectural Nursing Universtiy, 1-1, Gakuendai, Kahoku, Ishikawa 929-1212 Japan; Kitasato University, School of Nursing, 2-1-1, Kitasato, Sagamihara, Kanagawa 228-0829 Japan

**Keywords:** Older adults, Community-dwellers, Steps per day, Body fat percentage, Physical activity, Walking, Climate

## Abstract

**Background:**

An increasing number of older adults require improvements in their quality of life. Physical activities, particularly walking ability, are of primary importance for older adults. The influence of season on physical activity has not been sufficiently studied among older adults. Therefore, this report compared the physical activity and walking of older individuals between summer and winter seasons using a longitudinal study design in a community in a mid-latitude area.

**Methods:**

Participants in the study comprised 39 healthy community-dwelling adults ranging in age from 65 to 80 years. Physical parameters and activities as well as the preferred speed of walking were measured at half-year intervals.

**Results:**

Significant seasonal differences from summer to winter and from winter to summer were detected. Specifically, body fat percentage, single-leg stance, walking speed, cadence, stride length, and trunk and head-trunk pitch ranges were greater in winter than in summer, whereas grip strength and steps per day were greater in summer. Temperature and total activity level were considered to be related to body fat percentage. Grip strength was thought to be affected by outdoor temperature. The possibility of relationships between increased activity per unit time in older adults and increased preferred walking speed, cadence, and stride length in winter temperatures was discussed.

**Conclusion:**

The seasonal climatic environment of the geographic area of this study affected the activity level of the participants. These results indicate that seasonality should be considered when analyzing physical activity and walking in older adults.

## Background

Recent increases in the proportion of older adults around the world have led to increased attention being focused on methods for improving the quality of life of older adults. Maintenance of physical activity, particularly walking, is crucial for independent living by older adults. The growing interest in physical and walking activities for older adults has resulted in the publication of many recent investigations [1–6]. However, relatively few studies on physical activity and walking by older adults have also reported on the season or climatic environment of the study. The influence of seasonal climate change on physical activity and walking has recently been studied in mid-latitude areas for younger adults [7]. However, despite the likelihood that seasonal climate change has a large influence on the physical activity of older adults, few studies have investigated the effects of seasonal climate change in older adults. We therefore sought to obtain empirical data on these effects in older adults. Since aging is a longitudinal process, seasonal data for older adults should be analyzed in longitudinal studies, not in transverse studies. The difficulty of collecting longitudinal data for older adults has contributed to the scarcity of such studies [5, 8–13].

The present report was based on a longitudinal study of healthy community-dwelling older adults in a mid-latitude area and was intended to form the basis of an examination of the effects of seasonal changes on physical activity and walking in older adults. Part of the study has been reported elsewhere [13]. Two extreme seasons were chosen: mid-summer, from the middle of July to the beginning of August, and mid-winter, from the middle of January to the beginning of February. These seasons were selected to maximize any effects of seasonality. The study area was the snowy countryside of the Hokuriku District, Japan. Seasonal changes between summer and winter were analyzed and discussed with regard to physical activity and walking. We hypothesized that total daily activity would differ between seasons and that activity levels would influence the physical and walking parameters.

## Method

### Participants

Thirty-nine volunteers (22 women and 17 men) living in their own homes near the study venue of a gymnasium were recruited. The age range was 65 to 80 years, with a mean age (M_age_) of 70.7 years. None were inpatients of hospitals or residents of long-term health-care centers. All volunteers were active and healthy enough to visit the study venue independently. No participants with severe gait difficulties or a need for walking aids were included. Data for age, height, and weight of participants at the start of the study are shown in Table 1. Data were obtained for a summer to the following winter and for a winter to the following summer for each individual.

**Table 1:** Participants at the start of the study

	Number	Age (years)	Height (m)	Body mass (kg)
Total	39	70.7 ± 3.2	1.569 ± 0.090	59.3 ± 9.5
Female	22	70.0 ± 2.5	1.504 ± 0.048	54.5 ± 6.4
Male	17	71.7 ± 3.9	1.653 ± 0.051	65.4 ± 9.5

Mean ± SD

The longitudinal study continued for 7 years from the summer of July 2004 to the winter of February 2011. A set of two consecutive seasons for each individual was analyzed in the present study. The whole longitudinal study will be reported in the near future. To eliminate training effects, early consecutive trials of each individual were used. All study protocols were performed in accordance with the Declaration of Helsinki and approved by the Ethics Committee of Ishikawa Prefectural Nursing University (No. 59-1). Participants were informed about the purposes and procedures of the study and provided written informed consent prior to enrollment. They were free to not attend or cease participation in the study at any time.

### Procedure

As details of measurements have been described elsewhere [14], the measurement methods are briefly reported here. The basic physical parameters and performance measured were height (Digital stadiometer; Nihon Iryoki Kenkyujo, Tokyo, Japan); body mass and body fat percentage as measured by bioelectrical impedance via electrodes fitted to the soles of the feet (TBF-410 body fat analyzer; Tanita, Tokyo, Japan); body mass index; grip strength; and single-leg stance with eyes open. For gait analysis, participants were asked to walk at their preferred usual speed across a 7-m-long level wooden walkway in the gymnasium. They were barefoot and wore dark clothing with white ball markers 30 mm in diameter attached bilaterally at the following points: vertex; acromion (shoulder joint); radiale (elbow joint); trochanterion (hip joint); merion laterale (knee joint); supratarsale fibulare (ankle joint); and dorsal center of the interphalangeal joint of the big toe. Kinematics while walking the middle 3 m on the walkway were recorded from a distance of 6 m using two charge-coupled device video cameras (MotionMeter 250; Redlake, Tucson, AZ, USA) with a field rate of 60 Hz and a shutter speed of 1/300th of a second, and data were analyzed using motion-analysis software (FrameDias II; DKH, Tokyo, Japan). Only movements in the sagittal plane are reported in the present paper. Stride length and cycle duration were measured from the first contact of a foot on the floor to the next contact of the same foot. Axes of body segments in the sagittal plane were as follows: head-trunk axis, the line connecting the vertex and hip joint; trunk axis, the line connecting the shoulder and hip joints; upper arm axis, the line connecting the shoulder and elbow joints; thigh axis, the line connecting the hip and knee joints; shank axis, the line connecting the knee and ankle joints; and foot axis, the line connecting the ankle joint and big toe. Shoulder, hip, knee, and ankle joint angles were the angles between the trunk and upper arm axes, the trunk and thigh axes, the thigh and shank axes, and the shank and foot axes, respectively. To allow free walking, participants wore as little equipment as possible other than markers and an accelerometer, the results for which are not included here but part of which have been reported elsewhere [6]. All methods were noninvasive. The study duration for each individual was limited to less than half an hour. The research was performed mainly in the afternoon from about 13:30 to 15:00. From the day following the trial, participants were asked to wear a pedometer (Kenz Lifecorder EX; Suzuken, Nagoya, Japan) at their waist during waking hours for a week. Mean step count per day was calculated automatically.

To eliminate the effects of size differences, dimensionless values were calculated. Lower limb length was determined as the trochanteric height [15], and stride length was standardized to lower limb length to be dimensionless. Dimensionless cycle duration was standardized by multiplying by the square root of gravitational acceleration and dividing by the square root of lower limb length, by analogy with an inverted pendulum [16]. Dimensionless speed was then standardized by dividing by the square root of gravitational acceleration and the square root of lower limb length. Force was standardized to body weight. Kinematic data and grip strength were compared using the average of one trial each for the right and left sides of the individual. Single-leg stance duration ended at 60 s. The stance side was selected by the participant. Each participant performed two trials of stance and the best score was recorded. Statistical evaluation was applied to the percentage of single-leg stance (100 × (stance duration/60)) transformed to the natural logarithm.

As a statistical test, the paired *t* test was applied using Office Excel 2003 software (Microsoft Japan, Tokyo, Japan). Apparent seasonal differences were detected in the present study when differences between data of a summer and the following winter and between data of a winter and the following summer were both significant but inversed.

### Climatic environment

The city of Kahoku, where the study gymnasium is located (36° 46.8′ N, 136° 44.0′ E; altitude, about 35 m) [17], has a population of about 34,000, is in the Hokuriku region of Japan between the Sea of Japan and some low mountains, and is about 20 km from Kanazawa, the capital of Ishikawa Prefecture. Climate records for study days are shown in Table 2, as means and standard deviations (SDs), for 14 summer days and 14 winter days (two study days in each season) at the Kahoku Meteorological Station (36° 42.7′ N, 136° 41.5′ E; altitude, 42 m) (calculated by the authors using daily data from the Japan Meteorological Agency [18]) or at Kahoku City Hall (36° 43.2′ N, 136° 42.4′ E; altitude, converted to 0 m) [19]. Temperature differences between summer and winter were significant according to *t* testing with a difference of about 23 °C for each of the mean, maximum, and minimum temperatures. The gymnasium had no air-conditioning and was warmed by kerosene stoves in winter and cooled by electric fans in summer. Mean room temperature in the gymnasium at the study time at more than 4 m from stoves or fans was 30.3 ± 2.1 °C in summer and 10.4 ± 2.5 °C in winter, a few degrees higher than the maximum temperatures outdoors. Duration of sunshine and day length showed seasonal differences, but precipitation and maximum wind speed did not show any significant differences. Weekly climate data in Kahoku after the study day (the time during which participants wore pedometers), as the means for 14 weeks (two study weeks in each season) in summer and winter are also shown in Table 2 as daily averages. All weekly temperatures and day lengths were similar to those of study days, with significant seasonal differences. Weekly duration of sunshine, water fall, and maximum wind speed showed no significant seasonal differences. No data for snowfall were reported at Kahoku. Mean annual snowfall over the 7 years of study at nearby Kanazawa Local Meteorological Observatory (36° 35.3′ N, 136° 38.0′ E; altitude, 57 m) was 158.0 ± 88.5 cm, maximum depth of snow cover was 32.0 ± 20.8 cm, and mean number of snowy days was 57.1 ± 13.1 [19].

**Table 2:** Climatic data at Kahoku

	Study day		7 days after study day	
	*n* = 14		*n* = 98	
	Summer	Winter	Summer	Winter
Temperature (°C)				
Mean	25.9 ± 2.1	3.3 ± 2.7***	26.1 ± 2.1	3.1 ± 2.8***
Maximum	29.5 ± 2.7	6.6 ± 3.8***	29.8 ± 2.8	6.4 ± 3.7***
Minimum	22.8 ± 1.7	-0.3 ± 2.3***	22.8 ± 1.6	-0.1 ± 2.1***
Sunshine duration (h)	5.66 ± 4.40	1.91 ± 2.73*	5.55 ± 4.26	2.29 ± 2.60
Precipitation (mm)	1.14 ± 2.35	8.93 ± 9.37	7.83 ± 18.80	5.08 ± 7.23
Maximum wind speed (m/s)	6.15 ± 2.03	9.01 ± 3.24	5.36 ± 1.95	7.05 ± 2.99
Day length (h)^a^	14.2 ± 0.2	10.3 ± 0.3***	14.1 ± 0.2	10.4 ± 0.3***

Mean ± SD at Kahoku Meteorological Station

^a^At Kahoku City Hall

*p < 0.05; ***p < 0.001 significant difference between summer and winter

## Results

Significant seasonal differences are apparent in the parameters shown in Table 3. Parameters showing differences were as follows: body fat percentage; single-leg stance; speed (both absolute (m/s) and dimensionless); dimensionless stride length; ranges of trunk and head-trunk pitches around the fronto-horizontal axis (all large values in winter); dimensionless grip strength; steps per day; and absolute cycle duration (s) (all large values in summer). No parameters showed significant monotonous increases or decreases. No seasonal differences according to the present definition were observed in height, body mass, body mass index, absolute grip strength (kg), absolute stride length (m), dimensionless cycle duration, stance phase duration relative to cycle duration percentage, difference in cycle duration between right and left sides (s), or ranges of shoulder, hip, knee, or ankle joint angles.

**Table 3:** Seasonal differences

	Summer to winter			Winter to summer		
	(*n* = 39)			(*n* = 34)		
	Summer	Winter	*p* value	Winter	Summer	*p* value
Body mass (kg)	59.3 ± 9.5	59.7 ± 9.6	0.0850	66.0 ± 9.9	58.7 ± 9.2	0.0002
Body mass index	24.0 ± 2.4	24.2 ± 2.3	0.0557	24.2 ± 2.4	23.7 ± 2.2	0.0002
Body fat percentage (%)	24.8 ± 5.3	27.4 ± 6.0	<0.0001	27.8 ± 5.6	24.6 ± 5.0	<0.0001
Grip strength (body weight)	0.507 ± 0.103	0.464 ± 0.091	0.0001	0.450 ± 0.074	0.480 ± 0.093	0.0087
Single-leg stance (s)	41.9 ± 22.3	46.6 ± 15.8	0.0087	47.8 ± 15.2	42.6 ± 21.3	0.0027
Steps per day (steps)	8084 ± 3237	6098 ± 2625	<0.0001	6021 ± 2569	8581 ± 3397	<0.0001
Cycle duration (s)	1.013 ± 0.100	0.978 ± 0.091	0.0006	0.970 ± 0.085	1.007 ± 0.095	0.0048
Cycle duration (dimensionless)	3.54 ± 0.31	3.49 ± 0.33	0.1710	3.47 ± 0.33	3.57 ± 0.31	0.0228
Speed (m/s)	1.250 ± 0.144	1.306 ± 0.181	0.0084	1.319 ± 0.176	1.234 ± 0.153	0.0098
Speed (dimensionless)	0.447 ± 0.059	0.474 ± 0.067	0.0008	0.479 ± 0.065	0.447 ± 0.060	0.0082
Stride length (m)	1.255 ± 0.089	1.270 ± 0110	0.2325	1.274 ± 0.110	1.232 ± 0.088	0.0241
Stride length (dimensionless)	1.570 ± 0.137	1.638 ± 0.152	0.0023	1.645 ± 0.147	1.581 ± 0.145	0.0195
Trunk pitch range (degrees)	9.59 ± 2.36	10.64 ± 2.24	0.0139	10.72 ± 2.23	9.53 ± 1.92	0.0090
Head-trunk pitch (degrees)	6.01 ± 1.49	6.91 ± 1.81	0.0041	7.00 ± 1.71	6.19 ± 1.33	0.0042

Mean ± SD. Paired *t*-test

When each sex was evaluated separately, increasing and decreasing tendencies were the same as the overall results (data not shown), although the same parameters as in overall samples were not necessarily significant, probably because of the relatively small sample sizes. Women showed significant differences in body fat percentage and steps per day. Men showed significant differences in body mass, body fat percentage, body mass index, absolute grip strength, steps per day, dimensionless stride length, dimensionless speed, and ranges of trunk and trunk-head pitches.

**Fig. 1:**
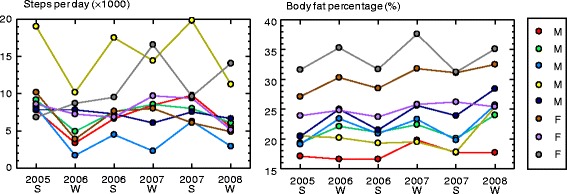
Examples of seasonal changes in the same individuals over three consecutive years. *Left*: steps per day; *Right*: body fat percentage (%). *S* summer, *W* winter, *F* female, *M* male. Values for the same individual are given using the same symbol to show seasonality. Steps per day and body fat percentage generally varied seasonally but in opposite directions

As representative examples of seasonality, Fig. 1 shows 3 years of continuous measurements of steps per day and body fat percentage in the same individuals. Although some individual variations were seen, steps per day and body fat percentage generally increased and decreased seasonally but inversely.

## Discussion

Smaller values for steps per day in winter, which were observed even when each sex was analyzed separately, indicated lower total daily activity compared to summer. This can be attributed to seasonal changes in lifestyle caused by the climatic environment. Togo et al. [5] observed clear seasonal changes in steps per day from over a 450-day period of longitudinal research on older adults (M_age_ = 71 years) living in the town of Nakanojo in Gunma Prefecture, Japan. Steps per day tended to increase with day length and mean ambient temperature within a range of −2 to 17 °C but decreased over the range of 17 to 29 °C. Mean temperature correlated with steps per day in mid-summer and mid-winter. Steps per day peaked in spring and/or autumn and reached the minimum value in winter. In summer, daily step counts were approximately the average for the year [9]. Daily activity counts were reported by means of the accelerometer over a 1-week period in a transverse study on older adults (over 65 years old, M_age_ = 76.5 years) in Tayside, Scotland [8]. Day length, maximum temperature, and duration of sunshine were able to explain 73 % of monthly variance in daily activity levels, and these three parameters represented independent predictors of daily activity in the study. All these parameters mentioned were smaller in winter than in summer at the present study site. Moreover, many snowy days and snow cover in winter would act to reduce total daily activity, especially outdoor activity, in the present district.

Seasonal variability in steps per day and body fat percentage was analyzed in adults in the UK between 18 and 65 years old [20]. Significant summer-to-following-winter reductions in steps per day and increases in body fat percentage were observed. The present study clearly demonstrated seasonal differences in both body fat percentage and ambulatory activity in older adults between 65 and 80 years old. Although some fluctuations were seen, especially in steps per day (Fig. 1), it is reasonable that some participants would encounter some reasons for short-term changes in activity level during the study days, not only due to physical or climate factors but also due to socio-cultural factors such as the presence of a special social activity in the specified season. Our results showed that a tendency still remained for the whole group. The significantly greater body fat percentage in winter, which was observed in each sex, would be related to the climatic condition of low temperature and decreased total daily activity. We can show some characteristics of seasonal differences in physical activity and walking.

Muscle activity in handgrip reportedly changes with skin temperature [21]. On the other hand, Krause et al. [22] reported no effect of 20-min cooling or 10-min warming of the skin on the amplitude of surface electromyograms of the forearm, although changes were seen in frequency. Because room temperature of the gymnasium changed between summer and winter, changes in grip strength could have been influenced by changes in temperature. Since changes in room temperature paralleled changes in outdoor temperature, the present results probably showed seasonal differences in grip strength in the environment of nearly outdoor temperatures.

Increases in walking speed and stride length and decreases in cycle duration (that is, increases in cadence) were observed in winter. Goodwin et al. [23] observed that activities per minute as measured at the wrist were larger in winter than in summer in a transverse study of older adults in the UK (M_age_ = 73.6 years). In the same investigation, controls comprising young adults (M_age_ = 23.5 years) showed no seasonal differences. The difference was attributed to older adults becoming more active per unit time in winter as a reaction to a reduction in body temperature. There was a possibility that high unit time activity caused the high speed, cadence, and stride length. The same tendency toward higher activity per unit time could increase ranges of trunk and head-trunk pitches in winter. These tendencies can be especially important to older adult activity in environments of nearly outdoor temperature. Details of the problem remain to be elucidated in future studies.

All participants in the present study were volunteer community-dwelling older adults with the physical strength and motivation necessary to visit the study venue. The measured parameters were relatively large or around the same as those reported for recent older Japanese adults in terms of height, body mass, body mass index, grip strength, single-leg stance duration, and steps per day [1, 10, 12, 24–26], although most reports were not recorded during the study season. Preferred speed was higher in the present study than that reported for the village of Nangai (present-day city of Daisen), Akita Prefecture, Japan [10, 12] but slower than that in Nakanojo [1], although the reported speed in the latter study was extraordinarily high, higher than that of young Japanese adults [14]. A key limitation of the present study was that the participants represented a kind of healthy “elite” [27].

Since force plates or behavioral interviews were not included in our study, our findings are limited by the lack of analyses from biomechanical or behavioral perspectives. Therefore, future studies should include such detailed analyses.

Clear seasonal differences were observed in many physical activities and walking parameters among healthy older adults, at least between summer and winter seasons, at the site of the present study. Seasonal climate differences influenced not only total daily activities of older adults but possibly also activity per unit time. Comparative physical studies of older adults should thus consider the effects of seasonal changes in the climate environment.

## Abbreviation

M_age_: mean age (years)
